# Deciphering the Genetic Blueprint behind Holstein Milk Proteins and Production

**DOI:** 10.1093/gbe/evu102

**Published:** 2014-05-14

**Authors:** Hyun-Jeong Lee, Jaemin Kim, Taeheon Lee, Jun Kyu Son, Ho-Baek Yoon, Kwang-Soo Baek, Jin Young Jeong, Yong-Min Cho, Kyung-Tai Lee, Byoung-Chul Yang, Hyun-Joo Lim, Kwanghyeon Cho, Tae-Hun Kim, Eung Gi Kwon, Jungrye Nam, Woori Kwak, Seoae Cho, Heebal Kim

**Affiliations:** ^1^Division of Animal Genomics and Bioinformatics, National Institute of Animal Science, Suwon, Republic of Korea; ^2^Department of Agricultural Biotechnology and Research Institute of Population Genomics, Seoul National University, Seoul, Republic of Korea; ^3^Interdisciplinary Program in Bioinformatics, Seoul National University, Seoul, Korea; ^4^CHO&KIM Genomics, SNU Research Park, Seoul National University Mt.4-2, Seoul, Republic of Korea; ^5^Division of Dairy Science, National Institute of Animal Science, Suwon, Republic of Korea; ^6^Division of Animal Biotechnology, National Institute of Animal Science, Suwon, Republic of Korea; ^7^Division of Animal Breeding & Genetics, National Institute of Animal Science, Cheonan, Republic of Korea

**Keywords:** Holstein, Hanwoo, domestication, milk protein, milk production, positive selection

## Abstract

Holstein is known to provide higher milk yields than most other cattle breeds, and the dominant position of Holstein today is the result of various selection pressures. Holstein cattle have undergone intensive selection for milk production in recent decades, which has left genome-wide footprints of domestication. To further characterize the bovine genome, we performed whole-genome resequencing analysis of 10 Holstein and 11 Hanwoo cattle to identify regions containing genes as outliers in Holstein, including *CSN1S1*, *CSN2*, *CSN3*, and *KIT* whose products are likely involved in the yield and proteins of milk and their distinctive black-and-white markings. In addition, genes indicative of positive selection were associated with cardiovascular disease, which is related to simultaneous propagation of genetic defects, also known as inbreeding depression in Holstein.

## Introduction

Understanding the forces that govern changes in populations or species over time is important for population genetics. Elucidating the relative contributions of genetic drift and natural selection to extant patterns of genetic variation is also important. As opposed to the neutral theory, a significant proportion of variation is believed to affect the ability of an organism to survive and reproduce, and will therefore be subject to natural selection (Kimura 1985; Gillespie 1991).

The rapid development of large-scale catalogs of genetic variation has increased interest in identifying targets of positive selection, which will ultimately increase our understanding of the roles of drift and selection during adaptation. Furthermore, signatures of positive selection delimit regions of the genome that are (or have been) functionally crucial. Therefore, identifying such regions will allow us to detect genetic variation that contributes to phenotypic diversity and facilitate functional annotation of the genome. In addition to these benefits, each target of positive selection can be used to characterize the historical forces and events that have shaped the history of a population (Biswas and Akey 2006).

For example, in humans, strong genetic signals of selection revealed several genes affecting skin, hair, or eye pigmentation (Lamason et al. 2005; Miller et al. 2007; Norton et al. 2007; Pickrell et al. 2009); genetic predisposition to high-altitude adaptation in Tibetans (Simonson et al. 2010; Yi et al. 2010); *LCT* for lactose tolerance or intolerance; and malaria resistance (Hamblin et al. 2002). The power of genome-wide analyses is not limited to humans; instead, several studies have been performed in domesticated species (Biswas and Akey 2006). As an example, genes associated with breed-specific characteristics—including varying sizes, shapes, colors, and temperaments—were investigated in domesticated dogs (Pollinger et al. 2005). Recently, a scan of the sheep genome for selection signals revealed 31 regions containing genes for coat pigmentation, skeletal morphology, body size, growth, and reproduction (Kijas et al. 2012). Additionally, Flori et al. identified 13 highly significant regions subjected to strong and/or recent positive selection by smoothing *F*_st_ values over each chromosome to explore the role of *GHR* in milk production and *MC1R* for coloration in three cattle breeds (Flori et al. 2009; Hayes et al. 2009).

Holstein cattle have been intensively selected during recent centuries, especially in the last few decades after the implementation of progeny-test-based breeding programs in the 1960s (Skjervold and Langholz 1964). Therefore, a number of breeds have become highly specialized for milk or beef production following strong artificial selection for these traits. This recent history provides a unique opportunity for the identification of loci subjected to adaptive selection (Brotherstone and Goddard 2005; Flori et al. 2009); the strong artificial selection will have increased the frequency of favorable alleles at the loci affecting milk production traits in the specialist milk production breeds (Smith and Haigh 1974). Identifying genome regions that have been subject to such “selective sweeps” in dairy cattle (but not in beef cattle) could reveal mutations responsible for increased milk production (Hayes et al. 2009).

In this study, we identified signals of selection based on a population comparison using the cross-population extended haplotype homozygosity test (XP-EHH), which was designed to detect ongoing or nearly fixed selective sweeps by comparing haplotypes from two populations (Sabeti et al. 2007; Tang et al. 2007). The extent and pattern of linkage disequilibrium (LD) between closely spaced markers contain information on population history, including past population size and selection history (Slatkin 1999). Although methods relying on LD, which breaks down rapidly over time, provide weak power to detect historical sweeps that are “ancient” (Chen et al. 2010), this specific research is exempt from these limitations as we consider recent selection events in Holstein cattle. Moreover, as few population genomics studies based on next-generation sequencing data are available, this study will advance our understanding of the mechanisms underlying Holstein characteristics. Thus, we analyzed the population statistics of two cattle breeds with different breeding histories to characterize the genetic basis of milk production traits in the Holstein breed, which resulted from recent and strong artificial selection.

## Materials and Methods

### Ethics Statement

All animals were handled in strict accordance with good animal practice, as defined by the relevant national and/or local welfare bodies, and all animal work was approved by the Institutional Animal Care and Use Committee of the National Institute of Animal Science (No. 2012-C-005).

### Samples and DNA Resequencing Data

Whole-blood samples (10 ml) were collected from 10 Holstein and 11 Hanwoo cattle. Holstein samples originated from different heifers inseminated with semen imported from Canada. We generated pair-end reads using Illumina HiSeq2000. DNA was isolated from whole blood using a G-DEXTMIIb Genomic DNA Extraction Kit (iNtRoN Biotechnology, Seoul, Korea) according to the manufacturer’s protocol. We randomly sheared 3 µg of genomic DNA using the Covaris System to generate inserts of approximately 300 bp. The fragments of sheared DNA were end-repaired, A-tailed, adaptor ligated, and amplified using a TruSeq DNA Sample Prep. Kit (Illumina, San Diego, CA). Paired-end sequencing was conducted with NICEM (National Instrumentation Center for Environmental Management, Seoul, Korea) using the Illumina HiSeq2000 platform with TruSeq SBS Kit v3-HS (Illumina). Finally, sequence data were generated using the Illumina HiSeq system.

We performed a per-base sequence quality check using the fastQC software (http://www.bioinformatics.bbsrc.ac.uk/projects/fastqc/, last accessed October 28, 2013). The pair-end sequence reads were then mapped against the reference bovine genome (UMD 3.1) using Bowtie2 (Langmead and Salzberg 2012). We used default parameters (except the “–no-mixed” option) to suppress unpaired alignments for paired reads. The overall alignment rate of reads to the reference sequence was 97.6% with an average read depth of 12.0× (8.77× to 14.77×). On average across the whole samples, the reads covered 99.23% of the genome (supplementary table S1, Supplementary Material online).

We used open-source software packages for downstream processing and variant calling. Using the “REMOVE_DUPLICATES=true” option in “MarkDuplicates” command-line tool of Picard (http://picard.sourceforge.net, last accessed November 15, 2013), potential PCR duplicates were filtered. We then used SAMtools (Li et al. 2009) to create index files for reference and bam files. Genome analysis toolkit 1.4 (GATK) (Nekrutenko and Taylor 2012) was used to perform local realignment of reads to correct misalignments due to the presence of indels (“RealignerTargetCreator” and “IndelRealigner” arguments).

The “UnifiedGenotyper” and “SelectVariants” arguments of GATK were used for calling candidate single nucleotide polymorphisms (SNPs). To filter variants and avoid possible false positives, argument “VariantFiltration” of the same software was adopted with the following options: 1) SNPs with a phred-scaled quality score of less than 30 were filtered; 2) SNPs with MQ0 (mapping quality zero; total count across all samples of mapping quality zero reads) >4 and quality depth (unfiltered depth of nonreference samples; low scores are indicative of false positives and artifacts) less than 5 were filtered; and 3) SNPs with FS (phred-scaled *P* value using Fisher’s exact test) >200 were filtered as FS represents variation on either the forward or the reverse strand, which are indicative of false-positive calls.

We used BEAGLE (Browning and Browning 2007) to infer the haplotype phase and impute missing alleles for the entire set of cattle populations simultaneously. A summary of the total number of SNPs, distribution of quality score, and chromosomal distribution of SNP densities in each 1-Mb bin are provided in supplementary table S2, figures S1 and S2, Supplementary Material online, respectively. Sequences are available from GenBank with the Bioproject accession numbers PRJNA210521 (Holstein) and PRJNA210523 (Hanwoo).

### Genotype Concordance

We additionally genotyped all cattle samples using BovineSNP50 Genotyping BeadChip (Illumina, Inc.). After filtering out SNPs based on a missingness rate >1%, minor allele frequency < 0.05, and Hardy–Weinberg equilibrium test *P* value < 10^−6^, common loci of SNP chip and DNA resequencing data were extracted and examined to assess concordance. SNPs were pruned using PLINK (Purcell et al. 2007). We observed 97.57% genotype concordance (supplementary table S3, Supplementary Material online).

### Statistics to Explore Selective Sweep Regions

The method cross-population extended haplotype homozygosity (XP-EHH) was first used to detect selective sweeps using the software xpehh (Sabeti et al. 2007) (http://hgdp.uchicago.edu/Software/, last accessed June 3, 2014). For each SNP loci, we calculated EHH and the log-ratio iHH (integrated EHH) for the pairwise test of the Holstein and Hanwoo populations. An XP-EHH score is directional: An extreme positive score implies selection in Holstein, whereas a negative score suggests selection in the Hanwoo population. The log ratios were standardized to have a mean of 0 and variance of 1. An XP-EHH raw score distribution plot is provided in supplementary figure S3, Supplementary Material online. We then split the genome into nonoverlapping segments of 50 kb to use the maximum (positive) XP-EHH score of all SNPs within a window as a summary statistic for each window. To take into account the SNP frequency, we binned genomic windows according to their numbers of SNPs in increments of 200 SNPs (combining all windows ≥ 600 SNPs into one bin). A histogram of SNP density in each window is provided in supplementary figure S4, Supplementary Material online. Within each bin, for each window *i*, the fraction of windows with a value of the statistic greater than that in *i* is defined as the empirical *P* value, following the method previously reported (Pickrell et al. 2009; Granka et al. 2012). The regions with *P* values less than 0.01 (1%) were considered strong signals in Holstein. In this report, the “*P* values” denote empirical *P* values; a low *P* value indicates that a locus is an outlier with respect to the rest of the genome. This approach is suitable, especially when the demographic parameters are unreliable and an explicit demographic model cannot be defined (as is the case for cattle) (Pickrell et al. 2009). However, loci detected as being under selection using this approach may be an underrepresentative sample of all truly selected loci; in particular, selection on standing variation and recessive loci are likely underrepresented (Teshima et al. 2006).

We additionally performed the cross-population composite likelihood ratio test (XP-CLR) for detecting selective sweeps that involves jointly modeling the multilocus allele frequency between two populations (Chen et al. 2010). XP-CLR scores were calculated using scripts available at http://genepath.med.harvard.edu/∼reich/, last accessed June 3, 2014. We used the parameters as the following: Nonoverlapping sliding windows of 50 kb, maximum number of SNPs within each window as 400, and correlation level from which the SNPs contribution to XP-CLR result was down weighted 0.95. The regions with the XP-CLR values in the top 1% of the empirical distribution (XP-CLR > 282.3) were designated candidate sweeps.

“Significant” genomic regions identified from XP-EHH and XP-CLR were annotated to the closest genes (UMD 3.1). Genes that span (partially or completely) the window regions were defined as candidate genes.

### Breeds and Sample Size

The XP-EHH approach requires a second population, carefully selected so that the two populations do not have signal overlaps that could hide some of the selection areas in the population of interest. Korean beef producers have selected Hanwoo for meat yield and quality (Chung and Kim 2005); a completely different breeding history against Holstein cattle will reveal the greatest selection pressure for the overall breeding goal in dairy cattle (supplementary fig. S5, Supplementary Material online). The Holstein population has been subjected to more than 50 years of intense selection for milk production traits (Oltenacu and Algers 2005). This recent selection history allows us to apply XP-EHH analysis. The time frame of approaches in detecting selection in genome-wide selection studies varies greatly, and the test based on extended LD segments is suitable for the most recent selection (Oleksyk et al. 2008). In addition, so long as the second population has a fixed sample size, XP-EHH maintains power with as few as 20 chromosomes (Pickrell et al. 2009), indicating that our study experienced minimal power loss.

### Coalescent Simulation under Demographic Models

Coalescent simulations were performed using the software “ms” (Hudson 2002). As the detailed genetic structure and history of cattle are not known (Bovine HapMap Consortium 2009), demographic events were investigated through four scenarios: Neutral, bottleneck, strong selection, and weak selection models. The number of segregating sites was set to 100 (-s 100), and 1,000 data sets were simulated under each scenario considered. For all simulations, the number of chromosomes sampled was 42 (20 Holstein and 22 Hanwoo), and we assumed the mutation parameter θ = 0.0012, generation time = 5 years, and effective population size N*_e_* = 300, following the literature (Bovine HapMap Consortium 2009; Murray et al. 2010; Pérez-Enciso 2014). Divergence time between Holstein and Hanwoo is not known; considering that Hanwoo was migrated and settled in the Korean Peninsula in BC 4000 years ago (Rhee and Kim 2001), we assumed the split time to be roughly 6 ka (1,200 generations). Scaling is in units of 4*N*_e_ generations. We further simulated data with this “neutral” demographic model under different conditions. For a bottleneck model, a bottleneck reducing the population size occurred in Holstein from 40 to 36 ka with intensity of 0.01 (Murray et al. 2010; McTavish 2013). For scenarios with selection, simulations were implemented with the software “msms” (Ewing and Hermisson 2010). We assumed that the selection started ten generations before present (50 years ago) (Qanbari et al. 2010). The selection strength was set to 100 and 500 (in weak and strong selection model, respectively), and the selection intensity for the advantageous homozygote was assumed to be twice of the intensity of the heterozygote. For all SNPs in the simulation regions in each model, we calculated XP-EHH scores to compare the distribution. A summary of parameters are described in supplementary table S4, Supplementary Material online.

### Population Differentiation

**Fig. 1.—:**
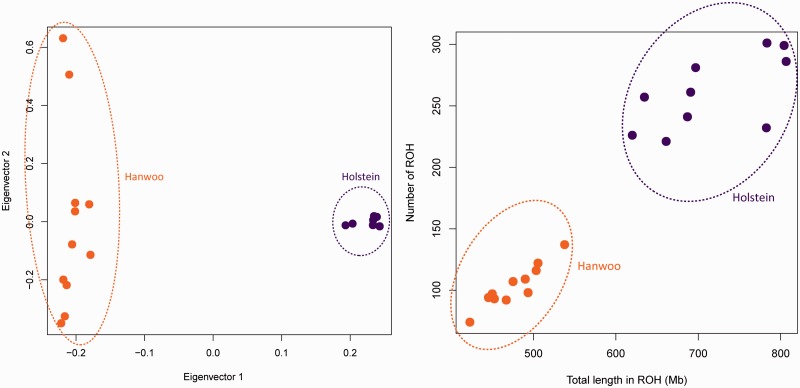
PCA results and individual patterns of long runs of homozygosity of the Holstein/Hanwoo samples. Eigenvector 1 against eigenvector 2 from PCA is plotted (*A*). The proportions of variance that explained the two most informative. eigenvectors were 13.6% and 6.4%. The number of runs of homozygosity was compared with the total length in ROH for Holstein and Hanwoo populations (*B*). Samples are color coded to indicate each breed.

We used VCFtools 4.0 (Danecek et al. 2011) to define the long run of homozygosity (LROH) and to estimate nucleotide diversity. We filtered out homozygosity segments shorter than 50 kb for LROH analysis (fig. 1). For principal component analysis (PCA), we used the genome-wide complex trait analysis (GCTA) (Yang et al. 2011) to estimate the eigenvectors, which is asymptotically equivalent to those from the PCA implemented in EIGENSTRAT (Price et al. 2006), incorporating genotype data from 10 Holstein and 11 Hanwoo samples. For admixture analysis, we restricted the genotype data to a random subset of approximately 0.1% of total SNPs using PLINK (–thin option) (Purcell et al. 2007) to run the “admixture” model with *K* = 2 in STRUCTURE version 2.3 (Hubisz et al. 2009). We chose 20,000 iterations after a burn-in of 100,000 iterations. Using the same restricted genotype data, Treemix 1.12 (Pickrell and Pritchard 2012) was used to determine the historical relationship between two populations and to check for migration events with 1,000 bootstraps replicated. We allowed potential migration events (-m flag) in the model. Treemix models the genetic drift at genome-wide polymorphisms to infer relationships between populations.

### Genes as Outliers in Holstein Cattle

We used the Database for Annotation, Visualization and Integrated Discovery (DAVID) version 6.7 to analyze the KEGG pathway (Huang et al. 2008). Milk production trait quantitative trait loci (QTL) from the animal QTL database (Animal QTLdb) were defined by a trait class of “Milk,” which consisted of 2,057 QTL (Hu and Reecy 2007).

## Results and Discussion

### Sequencing, Assembly, and Identification of SNPs

The genomes of 10 Holstein and 11 Hanwoo were sequenced to 12.0× coverage on average, with a total of 4.75 billion reads comprising approximately 665 Gbp. Using Bowtie 2 (Langmead and Salzberg 2012), reads were aligned to the reference genome sequence UMD 3.1 with an average alignment rate of 97.6%, that covered 99.23% of the genome (supplementary table S1, Supplementary Material online). After filtering the potential PCR duplicates and correcting for misalignments due to the presence of INDELs, we detected autosomal SNPs using GATK (Nekrutenko and Taylor 2012). Several filtering steps were applied before using candidate SNPs for further analyses to minimize the number of false-positive calls. We removed SNPs based on the following criteria: Phred-scaled quality score, mapping quality, quality depth, and phred-scaled *P* value. We finally retained a total of approximately 17.7 million autosomal SNPs (supplementary table S2, Supplementary Material online). The same samples were additionally genotyped with Illumina BovineSNP50 BeadChip to cross-validate the genotype calls from resequencing data. We observed 97.57% overall concordance between SNPs detected by BovineSNP 50 and resequencing (supplementary table S3, Supplementary Material online).

### Population Structure

We performed PCA of the autosomal genotype data from Holstein and Hanwoo samples (fig. 1*A*) using GCTA (Yang et al. 2011), which implemented EIGENSTRAT (Price et al. 2006). Global patterns of genetic structure can be inferred by PCA. The analysis ignores breed membership but revealed clear structures as samples from the same breed clustered together. The largest principal component (13.6% of the total variation) positioned Holstein apart from Hanwoo samples. The samples showed no evidence of admixture with each other.

When an individual’s parents share a relatively recent common ancestor, large portions of their genomes will be identical-by-descent. If both parents transmit the same segment to the child, the child will be homozygous for that segment, thus creating a run of homozygosity (ROH) (McQuillan et al. 2008). There is a continuum of homozygous segment length depending on the degree of shared parental ancestry and age. ROH due to recent inbreeding will tend to be longer as there has been little opportunity for recombination to break up the segments that are identical-by-descent. Therefore, it is of interest to compare the extent of homozygosity between populations with different degrees of isolation and consanguinity (Kirin et al. 2010). The complement of ROH in an individual genome may be represented efficiently by plotting the number of ROH against the total length (fig. 1*B*). The same trends can be found across breeds. Samples from breeds tend to cluster on the plot with regard to both the total length and the number of ROH. In addition, we observed that Holstein (compared with Hanwoo) had longer and more frequent runs of homozygosity as a response to the rapid artificial selection.

To understand the degree of admixture in the populations more deeply, we used STRUCTURE (Hubisz et al. 2009) on a randomly sampled subset of 17,247 SNPs (∼0.1% of the total SNPs). As in supplementary figure S6, Supplementary Material online, admixture between Holstein and Hanwoo was merely shown. In addition, the Treemix analysis detected no potential migration events between any pairs of populations (supplementary fig. S7, Supplementary Material online). Migration events can be expressed as edges in the tree and are colored according to their weight; yet there was no evidence of migration between Hanwoo and Holstein in our analysis.

### Genomic Regions with Selection Signals

To pinpoint loci under positive selection, we calculated the XP-EHH statistic between Holstein and Hanwoo population groups. This statistic assesses haplotype differences between two populations and is designed to detect alleles that have increased in frequency to the point of fixation or near-fixation in one of the populations (Sabeti et al. 2007; Pickrell et al. 2009). A comparison between Holstein and Hanwoo samples is appropriate because these populations have historically lived under different environments.

To study the robustness of the approach, we first investigated the null distributions of XP-EHH scores for different demographic models. A total of four demographic scenarios between Holstein and Hanwoo populations were tested, and samples were generated by coalescent simulations. A total of 1,000 data sets were simulated in a range of demographic models including: Standard neutral model, bottleneck in Holstein population, relatively weak and strong selection pressure in Holstein. We observed that the distribution of XP-EHH scores closely matched regardless of the demography (supplementary fig. S8, Supplementary Material online). Based on this, and from previous studies (Moreno-Estrada et al. 2009; Pickrell et al. 2009), we hypothesized that XP-EHH scores can be robust to variation in demographic models. Given that there are no concrete and perfect models of cattle breed demographic history, we proceeded to identify a locus that is an outlier with respect to the rest of the genome, following the previous study (Pickrell et al. 2009).

While sampling large genomic SNPs, empirical distributions can be constructed and genes subjected to the local forces, such as selection can be identified using the outlier approach (Kelley et al. 2006). To facilitate comparisons of genomic regions across populations, we divided the genome into nonoverlapping segments of 50 kb and computed the window statistic as the maximum XP-EHH score in each segment. In each window, we converted the test statistic to an empirical *P* value based on its ranking, taking into account the number of SNPs in the window. A set of regions that show evidence of local positive selection was identified by using an empirical significance level of 0.01.

The regions with outlier SNPs provide specific candidate regions for fine-scale mapping of genes that are important for Holstein cattle domestication. We identified major genes as outliers from a total of 250 genes from XP-EHH test (table 1 and supplementary tables S5 and S7, Supplementary Material online). The genes included *CSN1S1* and *CSN2* (*P* = 1.22E-03; XP-EHH = 5.17). These results suggested that milk-related traits of Holstein breeds resulted from local positive selection on several distinct genes. The milk protein genes alpha_s1_-casein (*CSN1S1*) and beta-casein (*CSN2*) are relevant to milk production parameters and milk protein quality (Kucerova et al. 2006). Such genes correlated with performance parameters explain a part of the genetic variance and can improve the estimation of breeding values. Therefore, they can be used to supplement conventional breeding procedures (Pribyl 1995). Besides the genes responsible for milk production traits, the genome-wide selection scan also identified a gene (*KIT* on BTA 6, *P* = 8.14E-05; XP-EHH = 5.95) associated with coat color. As opposed to the brown coat color of Hanwoo (Sasazaki et al. 2005), Holsteins have distinctive black-and-white markings, and white spotting of the coat is observed in numerous domesticated mammals. In horses and pigs, the *KIT* gene is commonly known for its association with a white spotting pattern and white coat color phenotypes (Moller et al. 1996; Haase et al. 2009). In domesticated cattle, the widely conserved KIT locus affects the degree of white spotting (Liu et al. 2009).

**Table 1:** Summary of Major Candidate Regions Identified from XP-EHH

Genes in XP-EHH Regions	Chromosome	Window (Mbp)	SNPs^a^	Max XP-EHH^b^	*P* value^c^
*KIT*	6	71.75–71.8	516	5.95	8.14E-05
*SULT1E1*	6	87.05–87.1	305	5.57	3.57E-04
*CSN1S1*, *CSN2*, *HSTN*	6	87.15–87.2	472	5.17	1.22E-03
*ITGAV*	2	9.65–9.7	181	3.48	9.30E-03

Note.—See supplementary table S8, Supplementary Material online, for descriptions of these major candidate genes and table S5, Supplementary Material online, for summary values of all 250 candidate genes.

^a^A total number of SNPs located within this window.

^b^Maximum (positive) XP-EHH score of all SNPs within a window.

^c^Rank-based empirical *P* value of genomic region.

We next compared our results with those of previous studies. We first observed that the majority of genes (242 out of 250, 96.8%) resided in QTL for milk production traits. In a previous study, Ron et al. generated the database of candidate genes for milk production traits in cattle (cgQTL) (Ron and Weller 2007). We identified five genes that were present in the cgQTL database: *ITGAV*, *CSRP1*, *ATP1A2*, *CASQ1*, and *RAB1A*. Larkin et al. (2012) recently reconstructed the haplotypes of two influential sires of the contemporary Holstein-Friesian population to identify 11 genes with SNPs that have been subjected to artificial selection for milk production, fertility, and disease-resistance traits. Of the 11 candidate genes, *SULT1E1* showed a concordant result with our study. In addition, gene families *ITGA6* and *BMP4* (*ITGAV* and *BMP10*, respectively) were also detected. Based on the database of cattle candidate genes and genetic markers for milk production and mastitis developed by Ogorevc et al. (2009), *CSN1S1* and *CSN2* have been frequently reported to be associated with milk production performance and mastitis; putative miRNA target sites in candidate genes expressed in mammary gland include *GLI3*, *ITGAV*, and *CSN2*. From the work of Lemay et al. (2009), *RAB10* and *RAB1A* were identified as milk protein gene set along with *CSN1* and *CSN2*.

### Combining Selection Signals

If each signature provides distinct information about positive selection, combining signals provide greater power for localizing the source of selection (Grossman et al. 2010). To search for regions in the genome where the change in allele frequency at the locus occurred too quickly due to random drift, we used the XP-CLR (Chen et al. 2010). All regions above a threshold of 282.3 (top 1% of the empirical distribution) can be considered significant, identifying 253 positively selected genes in Holstein compared with Hanwoo (supplementary tables S6 and S7, Supplementary Material online). We observed 62 genes in the intersection with XP-EHH selection candidates. The candidate regions included additional milk protein gene, kappa-casein (*CSN3*, XP-CLR = 325.78). Previous studies found a favorable effect of the CSN3 variant on protein yield and protein content (Van Eenennaam and Medrano 1991; Bovenhuis et al. 1992), and although conflicting, the variant of *CSN3* has been associated with higher milk yield (Van Eenennaam and Medrano 1991). The time frame of approaches in detecting selection in genome-wide studies differs such that statistics using changes in the shape of the frequency distribution between populations (e.g., XP-CLR) have good power to detect older signals compared with those using extended LD segments (such as XP-EHH) (Oleksyk et al. 2010). In addition, XP-CLR is more robust to selection from standing variation (Chen et al. 2010).

### Reduction in Nucleotide Diversity

Nucleotide diversity measures the degree of polymorphism within a population, and is defined as the average number of nucleotide differences per site between any two DNA sequences chosen randomly from the sample population (Nei and Li 1979). Numerous previous studies reported a reduction in nucleotide diversity levels after a recent episode of positive selection (Gilad et al. 2002; Haudry et al. 2007). We first observed that on a genome-wide scale of every 10 Mb, the Holstein breed showed reduced levels of nucleotide diversity compared with the Hanwoo breed (supplementary fig. S9, Supplementary Material online). The reduced level of nucleotide diversity at the whole-genome level in Holstein is indicative of genetic drift followed by a unique demographic history.

**Fig. 2.—:**
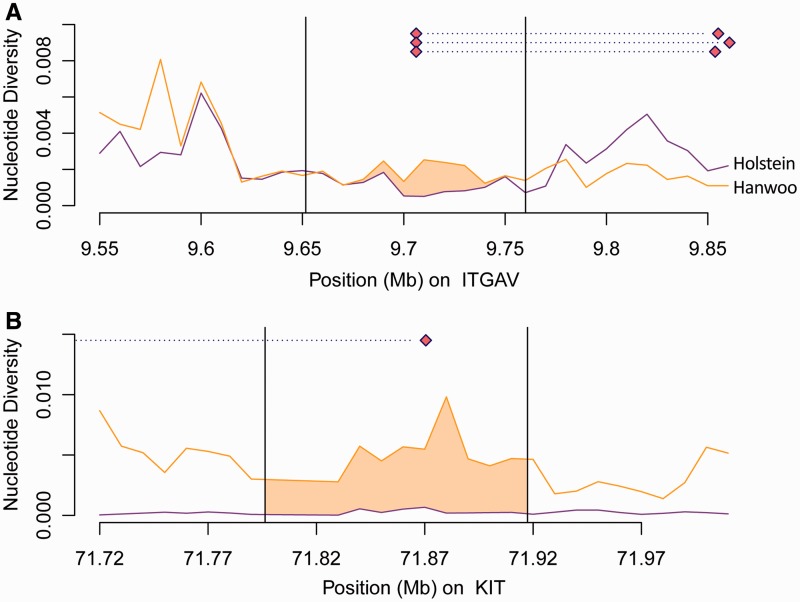
Nucleotide diversity plot of two major genes. The region shaded in orange represents the region in which the nucleotide diversity of Holstein (purple) is lower than that of Hanwoo (orange) at each position. The nucleotide diversity was estimated for each 10-kb window. Each dotted line represents the ROH segment of each Holstein sample within and near the candidate genes.

We next explored nucleotide diversity for each gene region. Of the positively selected genes from genome-wide scans, we manually defined six “major” candidates that showed overlaps with previous reports (supplementary table S8, Supplementary Material online). Although indiscriminate reduction of nucleotide diversity in Holstein at a genome-wide level was expected, the diversity between two populations was not always differentiable at the window size of 10 kb (supplementary fig. S10, Supplementary Material online). To distinguish between selection signatures and demographic effects from nucleotide diversity measure, we searched for genes that showed extreme reduction under diversity, especially in the gene region compared with the neighboring regions (fig. 2). The *ITGAV* gene showed a significant reduction in diversity, particularly at this gene site compared with the neighboring regions, where the diversity of Holstein and Hanwoo was indistinguishable. In addition, we observed a low diversity in Holstein as opposed to the increase in diversity in Hanwoo in *KIT*. These unique features of genetic diversity support the evidence of positive selection.

### Genes that May be Responsible for Inbreeding Depression in Holstein Cattle

We next analyzed the KEGG pathways, which revealed that genes as outliers in Holstein are involved in cardiovascular diseases including hypertrophic cardiomyopathy, dilated cardiomyopathy, and arrhythmogenic right ventricular cardiomyopathy. Three cardiomyopathy-associated genes (*ACTC1*, *ITGAV*, and *ITGA2*) were positively selected in Holstein. Although the increased frequency of alleles and the increase in milk yield are beneficial for animal production, the simultaneous association with economic traits, such as the propagation of genetic defects, is not (Agerholm et al. 1993). Because certain important cattle breeds are widely disseminated globally, defective genes are likely present in the Holstein population (Zenger et al. 2007). The most common mode of transmission of genetic defects in cattle is autosomal recessive inheritance (Windsor and Agerholm 2009), and dilated cardiomyopathy and several heart diseases have been reported in Holstein globally (Nart et al. 2004; Buczinski et al. 2010).

However, this study should be regarded as hypothesis-generating rather than hypothesis-testing. The identified genes have hypothetical relationships with milk yield and milk protein concentration. Phenomena other than selection, such as genetic drift or inbreeding, could also be responsible for some of the results. Genes inferred to be positively selected in multiple scans would reduce this uncertainty because they are more likely to be true selection signatures.

## Conclusion

Cattle are a striking example of variation under domestication, yet the evolutionary processes underlying the genetics of this diversity are poorly understood. Patterns of genetic variation are commonly used for the study of domestication, breed formation, population structure, and the consequences of selection. We examined the patterns of diversity across the whole genome of Holstein cattle using SNP resequencing data to identify genomic regions that have undergone dramatic changes in response to selection. The significant genes can be used to characterize functional variants and explore the specificity of the Holstein breed.

## Supplementary Material

Supplementary figures S1–S10 and tables S1–S8 are available at Genome Biology and Evolution online (http://www.gbe.oxfordjournals.org/).
